# Generating diversity and securing completeness in algorithmic retrosynthesis

**DOI:** 10.1186/s13321-025-00981-x

**Published:** 2025-05-13

**Authors:** Florian Mrugalla, Christopher Franz, Yannic Alber, Georg Mogk, Martín Villalba, Thomas Mrziglod, Kevin Schewior

**Affiliations:** 1 Bayer AG, Leverkusen, Germany; 2 Frankfurt, Germany; 3 Cologne, Germany; 4 Present Address: Department of Mathematics and Computer Science, University of Cologne, Cologne, Germany; 5 Department of Mathematics and Computer Science, University of Southern Denmark, Odense, Denmark

**Keywords:** Computer-Assisted Synthesis Planning (CASP), Retrosynthesis, DFPN, Chemical diversity score

## Abstract

**Abstract:**

Chemical synthesis planning has considerably benefited from advances in the field of machine learning. Neural networks can reliably and accurately predict reactions leading to a given, possibly complex, molecule. In this work we focus on algorithms for assembling such predictions to a full synthesis plan that, starting from simple building blocks, produces a given target molecule, a procedure known as retrosynthesis. Objective functions for this task are hard to define and context-specific. In order to generate a diverse set of synthesis plans for chemists to select from, we capture the concept of diversity in a novel chemical diversity score (CDS). Our experiments show that our algorithm outperforms the algorithm predominantly employed in this domain, Monte-Carlo Tree Search, with respect to diversity in terms of our score as well as time efficiency.

**Scientific Contribution:**

We adapt Depth-First Proof-Number Search (DFPN) (Please refer to https://github.com/Bayer-Group/bayer-retrosynthesis-search for the accompanying source code.) and its variants, which have been applied to retrosynthesis before, to produce a set of solutions, with an explicit focus on diversity. We also make progress on understanding DFPN in terms of completeness, i.e., the ability to find a solution whenever there exists one. DFPN is known to be incomplete, for which we provide a much cleaner example, but we also show that it is complete when reinforced with a threshold-controlling routine from the literature.

## Introduction

In organic chemistry, one of the most important problems consists in constructing a synthesis plan for a given molecule. Retrosynthesis constitutes a formal approach to this problem: One recursively deconstructs the molecule into building blocks that are readily available for purchase or easy to make. This reverse approach was originally introduced by Corey et al. in the late 1960 s, and it is by now a cornerstone technique in organic chemistry [1–3]. Yet, applying this technique in practice is a highly complicated task due to a large number of potentially relevant reactions as well as multiple constraints (building-block availability, functional-group protection, safety regulations, green-chemistry considerations), and it has traditionally required solid *human* expertise. In the last decade, however, the field of Computer-Assisted Synthesis Planning (CASP) underwent significant improvements driven by the successful combination of advances in machine learning and the availability of sufficient amounts of reaction data [4–11].

There are two predominant approaches for building a CASP tool, *template-based* and *template-free*. Template-based approaches encode reactions as the chemical landscape around a reaction center, which can be extracted either manually by experts or with a data-driven approach [5, 9]. These templates can be used during a retrosynthetic search in a recursive fashion to derive new intermediates and starting molecules of suitable complexity and availability. Template-free approaches, in contrast, do not rely on handcrafted or automatically extracted transformation rules. Following tools and approaches developed by the Natural Language Processing (NLP) community, a set of SMILES (words, reactants) is transformed into another set of SMILES (words, products) [12]. Coupling this one-step synthesis model with a powerful search algorithm has been shown to yield similar performance as the aforementioned methods [10].

In 2018, Segler et al. [9] showed the viability of a data-driven approach, using two neural networks: the first one to predict which transformation templates should be prioritized during the route search and the second one to grade them according to their plausibility. These advances were followed by copious research mostly concerned with the quality of these neural networks and how they ingest their input data, e.g., regarding the usage of transformation templates [5, 6, 10, 12–14].

This work considers the overall quality of the found synthesis plans more directly. The quality of a synthesis plan depends on a large number of aspects such as safety, yield, required level of expertise, and available laboratory equipment, whose levels of relevance may vastly differ depending on context. It is therefore extremely difficult to capture this concept in a formal definition. Our approach circumvents this issue and can be summarized as *quality through diversity*. That is, we aim to find not just a single synthesis plan but several of them, maximizing the diversity—a concept we formally define—in the set of plans that we generate. Chemists can then pick a synthesis plan or recombine chemical ideas present in the set of plans, to meet the criteria relevant in the context at hand.

This diversity is possible thanks to more than 250 years of chemical research leading to multiple known ways to synthesize a molecule, target compound, or intermediate from commercially available building blocks. Unfortunately, this also poses a selection problem in each retrosynthesis step due to the overwhelmingly large host of choices that has to be explored to find a single solution, let alone a diverse set of them. Multiple algorithms, many of them viewing retrosynthesis as a two-player game, were proposed to solve this problem [9, 15, 16]. The most commonly used one is Monte-Carlo Tree Search [6, 9, 17]. While this algorithm can reliably find multiple solutions, it is not clear whether this set of solutions turns out diverse.

Instead of MCTS, we consider Depth-First Proof-Number Search (DFPN) [18], a popular [19, 20] and more efficient variant of Proof-Number Search (PNS) [21]. A version of DFPN has also been successfully applied to retrosynthesis [16], but PNS and its variants just stop after finding the first solution. We introduce an adaptation to this algorithm which can be applied essentially to any version of PNS, whose output will be a set of solutions, with explicit focus on diversity. In the experimental part of our paper, we indeed find that our version, called DFPN*, generally outperforms MCTS with respect to our diversity score, but in fact also with respect to efficiently finding the first synthesis plan.

To make a case for DFPN and its variants, we also solve an open problem regarding it as a side result: It can be shown that (plain) DFPN is not *complete* [22], i.e., it is possible that there exists a solution, but DFPN runs into an infinite loop rather than outputting the solution. (We give a much smaller example showing that.) Refinements of DFPN that have seemed to be complete in experiments are df-pn(r) [23] and DFPN with the Threshold Controlling Algorithm (TCA) [24], but no proofs of completeness for such variants are known. We provide a proof of completeness for DFPN with TCA.

## Methods

In this section, we first discuss how to measure diversity, then how to reduce retrosynthesis to solving two-player games, then how to solve such games (especially through DFPN), and finally how to adapt DFPN to find sets of (diverse) solutions.

### Measuring diversity in pathways

Practicing chemists have a good intuitive notion of chemical diversity. However, it is challenging to formally define the diversity of a set of synthesis pathways fitting this intuition. Nonetheless, diversity is a key objective for current, actively used CASP tools. A broadly applicable metric is necessary to not rely on anecdotal observations [25] alone to track algorithmic changes towards higher diversity in said sets of synthesis pathways. Some efforts towards this where made before. A natural approach to the problem is to measure the diversity among the molecules [26, 27] appearing in the routes or simply count unique intermediates and building blocks. In Table 4 and Fig. 12 in the appendix, we provide the respective numbers for both algorithms studied here with respect to the search times. Such metrics are, however, not practical. Structural variations in reactants tend to be overemphasized as they may not alter the chemical nature of the reactions (e.g., different protection or leaving groups fulfilling the same purpose). Another approach to compare routes with each other is to calculate their graph edit distance or tree edit distance [28, 29]. A subsequent clustering on the corresponding distance matrix can be used to asses the diversity within a set of routes [30]. While the higher abstraction level allows for an increased focus on relevant difference between routes and therefore also improves the associated diversity metric, it also comes with its own drawbacks. First, the metric can go down by adding further routes to the set [31] which is, from a theoretical point of view, plausible but goes beside the point of gauging which set is more useful for lab practitioners. Second, while being a very elegant way of measuring distances between routes from a mathematical point of view, the graph edit distance is indiscriminate towards the importance of differences between routes from a chemical perspective. A very strict way to tackle the problem is to count only the number of routes with no overlap, as proposed by Maziarz et al. [31]. In our context, this is however too restrictive since potentially important chemical solutions can go unrecognized by the metric when paired with an already used reaction.

We have worked in close collaboration with several lab chemists from multiple different fields such as medicinal, agricultural, and process chemistry to formalize our approach to measure route diversity. The general understanding among lab chemists can be summarized as the number of different chemical ideas observed between the individual synthesis pathways. Unfortunately, chemical ideas are a similarly vague concept as diversity and therefore difficult to distinguish and count objectively.

For this reason we propose a new metric, dubbed Chemical Diversity Score CDS, based on the intuitive idea of disconnections (bond breakages) which was used in similar contexts before [10, 32]. The core idea is to mimic the thought process involved in a manual retrosynthesis analysis, making it quantifiable.

By identifying which bonds of a molecule get broken from the retrosynthesis point of view (i.e. formed in forward direction) in any given pathway, we keep track of the synthesis approaches present in a set of routes. We choose to focus on the final result of the route search (sets of full routes) over focusing on the diversity coming from the individual parts (like the reaction prediction model) of a given CASP tool. Model metrics do not necessarily have much bearing on the route search results (as demonstrated in Fig. 10 and Table 3) and gains achieved here might easily be overwritten by other parts of the pipeline like filtering mechanisms or the search algorithm itself. Figure 10 and Table 3 of Appendix D.5 lend support to our choice. Similar observations were made by Genheden and Bjerrum [29] and Maziarz et al. [31].

As a result, the higher chemical concept is still recognized while avoiding an artificially high diversity. For example, a famous and often used reaction type is the *Suzuki-coupling* [33]. There are about 20 variants of this technique to create the same carbon-carbon bond, which can lead to different synthesis routes, but which are all based on the same chemical idea. Based on our discussions with lab chemists, we consider the level of abstraction provided by the disconnections ideal, as they do not discriminate between different named reactions forming the same bond, but are still capturing different synthesis strategies.

To compute the $\text{CDS}$, all bonds formed in the proposed synthesis pathway get identified and labeled. From the resulting sets we select those which cannot be represented by another, smaller set of bonds found in all the pathways for the target molecule. This way, we eliminate miscellaneous reactions that do not contribute to the chemical diversity in a meaningful way, forming the set $C_M$. The final score is then obtained by calculating the mean over the all-to-all Jaccard distance matrix expressed by

$$\begin{aligned} \text {CDS}:= 1 + \frac{1}{|C_M|} \sum _{T \in C_M}\sum _{T' \in C_M} d_J(\hat{T},\hat{T}'). \end{aligned}$$

The $\text {CDS}$ can be interpreted as the number of different chemical ideas present in a given set of synthesis pathways. Higher $\text {CDS}$ values are considered better, as they indicate a higher diversity between them. A more detailed mathematical formulation and further explanations can be found in Appendix 4.1.

### Game-theoretical approaches to the retrosynthesis problem

Viewing retrosynthetic planning as games on a simple directed graph is a standard approach (e.g., [9, 16]). In the two-player game which we focus on in this paper, there is a node for every molecule and a node for every reaction. We will call one player *molecule player* and the other *reaction player*. The game starts at the node corresponding to the target molecule. It is the molecule (reaction) player’s turn whenever the game is at a molecule (reaction) node. The next node can be chosen according to the following directed edges: From a molecule node, the edges lead to all nodes corresponding to reactions having the molecule corresponding to the current node as product. From a reaction node, the edges lead to all nodes corresponding to reactants required for the reaction corresponding to the current node. The molecule player wins if a node corresponding to a building block is reached, and the reaction player wins if a dead end (i.e., a molecule that can neither be synthesized nor bought) is reached or a node is visited for the second time. This way, a winning strategy for the molecule player represents a synthesis plan for the target molecule. Conversely, if there exists a winning strategy for the reaction player, the target molecule cannot be synthesized with the reactions represented in the graph. For this emerging two-player game, PNS was first used in its regular form [15], whereas DFPN was first applied as part of the DFPN-E algorithm [16] and later within the CompRet tool [8]. In the next chapter, we will explain the (DF)PNS in more detail.

In what can be interpreted as a one-player game, a node usually corresponds to a set of molecules and each edge to a reaction. Monte-Carlo Tree-Search [9, 34] operates iteratively on such games, where each iteration consists of four steps: The first one is *selection*, where the most promising node gets selected. The second one is *expansion*, where the previously selected node gets expanded by creating one or more nodes. After that the *simulation* happens, where starting with these nodes a game to end nodes gets simulated, and lastly the *backpropagation*, where the result of these games is propagated back in the tree. Already visited nodes get penalized such that, when running for a long period of time, it can also find a set of different synthesis plans.

Very recently, Tripp et al. [35] use a type of greedy algorithm specifically designed for the task of creating multiple synthesis plans with a high probability that at least one of them is feasible, and called it Retro-fallback. Their heuristic can be summarized as simply expanding the molecule that is expected to have the highest increase of their so-called *successful synthesis probability* (SSP). For a given synthesis plan *T*, the SSP depends not only on the probability that *T* is successful, but also incorporates the probability of any of the previously found synthesis plans and whether *T* can significantly increase it. Other promising results were obtained using variations of A* search [4, 25].

### Depth-first proof number search and variants

To find winning strategies, *Proof-Number Search* (PNS) [21] explores the graph from the start node by iteratively expanding nodes that were previously not expanded. By doing so, PNS learns if the node is winning for one of the players (and if so, which) and explores its out-neighbors. A node is called *proved* if a winning strategy for the molecule player can be inferred from the explored part of *G*; if a winning strategy for the reaction player can be inferred, the node is called *disproved*. A node that is neither proved nor disproved is said to be *unproved*.

The *(dis)proof number* of an explored node *v* is defined to be the minimum number of explored unproved nodes that have to be (dis)proved for *v* to be (dis)proved. The precise numbers are NP-hard to compute [36]. PNS instead efficiently maintains estimates of these numbers, $\text{pn} (v)$ and $\text{dn} (v)$ for each node *v*; $\text{pn} (v)=\text{dn} (v)=1$ for unexpanded nodes *v* such as $v_0$ in the beginning. PNS uses $\text{pn} (\cdot )$ and $\text{dn} (\cdot )$ to determine the *most promising node*, the next node to expand: Starting from $v_0$, it iteratively selects the out-neighbor with the minimum proof number from a molecule-player node and with a minimum disproof number from a reaction-player node, until reaching a non-expanded node, which then is expanded. PNS then backtracks to $v_0$, updating $\text{pn} (\cdot )$ for a molecule-player node to be the minimum $\text{pn}$ value of an out-neighbor and for a reaction-player node to be the sum of these $\text{pn}$ values (vice versa for $\text{dn}$). If the graph is an out-tree, PNS exactly determines the proof and disproof numbers in this way.

Depth-First Proof-Number Search (DFPN) [18] does not start the search for the most promising node from $v_0$ in each iteration, making it more efficient. Instead, when node *v* is currently selected, it tries to use thresholds $\text{th}_{\text{pn}} (\cdot )$ and $\text{th}_{\text{dn}} (\cdot )$ to decide whether the path taken from $v_0$ to *v* is part of a path that PNS would take. In particular, if $\text{pn} (v)<\text{th}_{\text{pn}} (v)$ and $\text{dn} (v)<\text{th}_{\text{dn}} (v)$, then it continues the search like PNS would, and otherwise it backtracks one step. The values of $\text{pn}$ and $\text{dn}$ are only recomputed at the node that DFPN is currently considering. If *v* only has a single neighbor, the thresholds are simply passed on. Otherwise, if *v* is a molecule-player node, the $\text{th}_{\text{pn}}$ value of the chosen child becomes the minimum of the $\text{th}_{\text{pn}}$ value of *v* and the increment of the proof number of the second-best child. If *v* is a reaction-player node, the $\text{th}_{\text{pn}}$ value of the chosen child becomes the surplus between *pn*(*v*) and its threshold plus the $\text{pn}$ value of the chosen child. For $\text{dn}$, the operations are again analogous. As before, this is exact for out-trees, but DFPN is incomplete on general graphs [22].

As pointed out in [37], DFPN and PNS as stated above are not necessarily correct on graphs that contain cycles: A proof or a disproof of some node *v* that is found when *v* is visited through some path *P* cannot certainly be reused when *v* is visited through some path $P'\ne P$. This is called the Graph History Interaction Problem. As a general solution [23, 38], one can, upon (dis)proving a node when visiting it through *P*, save the (dis)proof only with respect to *P*. When visiting *v* again through $P'$, it can be verified if the previously found proof is still valid. For further details, we refer the reader to the aforementioned works.

To break infinite loops, the Threshold Controlling Algorithm (TCA) [24] maintains a value $\text{md} (v)$ for every node *v*. It represents the (minimum) distance between $v_0$ and *v* in the explored part of *G*. Whenever a node *v* is visited that has an unproved *old* child *c*, i.e., $\text{md} (c)\le \text{md} (v)$, TCA adjusts $\text{th}_{\text{pn}} (v)$ and $\text{th}_{\text{dn}} (v)$ such that DFPN does not backtrack. In particular, it sets $\text{th}_{\text{pn}} (v)$ and $\text{th}_{\text{dn}} (v)$ to values so that $\text{pn} (v)<\text{th}_{\text{pn}} (v)$ and $\text{dn} (v)<\text{th}_{\text{dn}} (v)$ are definitely satisfied. The fact that the thresholds have been increased is passed to subsequent recursive calls of the algorithm, prompting these calls to also increase the corresponding thresholds, until a node is expanded or a cycle is closed (i.e., progress is made).

We give a more formal description of the entire algorithm in a general context in Appendix A.

### Completeness of DFPN with TCA

We start this section with (re-)justifying variants of DFPN by giving a counter example to the completeness of DFPN that is significantly simpler than the known counter example [22]. In the second subsection we will then prove completeness of DFPN with TCA.

#### Simpler counter example without TCA

**Fig. 1:**
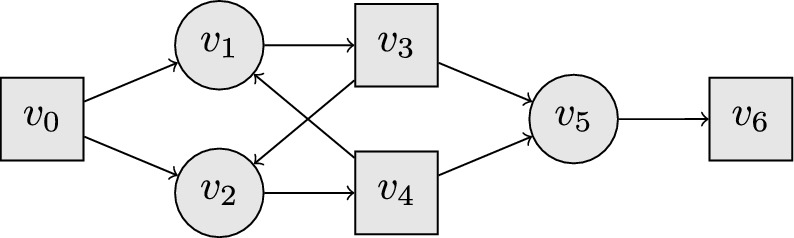
The graph *G* that shows that DFPN is not complete.The molecule nodes and the reaction nodes are depicted as squares and circles, respectively

We consider the graph shown in Fig. 1. In that graph, DFPN visits $v_3$ and $v_4$ alternating from $v_0$ via $v_2$ and $v_1$, respectively, in an infinite loop. In particular, it never expands $v_5$. When $W_0=\{v_6\}$ and $W_1=\emptyset$, DFPN therefore never discovers the only winning strategy for the molecule player from $v_0$, which includes $v_6$. In this example the TCA would upon visiting $v_3$ increase its threshold, since the minimal distance from its child $v_2$ to the root $\text{md} (v_2)$ is smaller than its $\text{md} (v_3)$. Therefore the algorithm would expand $v_5$ and find the winning strategy in $v_6$. In comparison to the known counterexample, this one only consists of 7 instead of 17 nodes and is much more symmetrical.

#### Proof of completeness with TCA

We show that DFPN with TCA is complete, that is, whenever there exists a winning strategy for the starting player, DFPN with TCA eventually finds such a strategy. In the overall structure, our proof resembles the proof that DFPN is complete on directed acyclic graphs [22]. In particular, we assume towards a contradiction that the algorithm gets into an infinite loop and consider the subgraph *L* of the entire graph that is relevant for the infinite loop. It can be seen that the infinite loop is due to inconsistencies of the $\text{pn}/\text{dn}$ values of the nodes in *L*. We manage to show that, as long as the algorithm stays in the infinite loop, the $\text{pn}/\text{dn}$ values become “less inconsistent” over time. Therefore, after finitely many steps, the algorithm will break the infinite loop.

We quantify inconsistency in the same way as Kishimoto and Müller [22], that is, by counting the number of inconsistent nodes in the different layers of *L* (defined according to the length of the longest path from the root) separately and collecting the counts in an inconsistency tuple. We show that, after finitely many steps, the inconsistency tuple decreases in a lexicographic sense.

The main additional technical ingredient is a fundamental property of *L* that is needed to prove the previous statement. Specifically, we consider a situation in which some node $n_c$ is searched, but the algorithm eventually backtracks because the threshold condition is not met any more. We show that, for any other node *m* at which the threshold condition has been violated in the meantime as well, there is no path from any higher-level node to *m*. This property helps us in controlling how inconsistencies can develop in *L*.

### Adaptation of DFPN to multiple solutions

Since the algorithm is deterministic, running it multiple times would only lead to the same solution each time. To find multiple solutions, we modify PNS and its variants. We change the values of $\text{pn}$ and $\text{dn}$ for some nodes in such a way that the found solution becomes invalid. We need to be careful about this for two reasons. First, the choice of nodes controls the type of diversity we obtain for the set of solutions we find when iterating this idea. Second, since we might end up at a node through different paths, we do not want to completely neglect any nodes. Instead, we keep track of the path we used to get to any node and store it. If we encounter the same node again, we check if the path that was used to reach it was stored. If so, the node will stay disproved, otherwise we can use it again.

To further control the diversity of the solutions, the algorithm penalizes every node used by a found winning strategy. This happens by adding a penalty£ $p_\text{reac}$ to the values of $\text{pn}$ for every reaction-player node *v* in a path to a node selected by the above diversity controlling strategy: $\text{pn} (v):=\text{pn} (v)+p_{\text{reac}}$. Adding a penalty $p_{\text{mol}}$ to the values of $\text{pn}$ for all molecule-player nodes *v* represents an additional approach. Afterwards the values of $\text{pn}$ and $\text{dn}$ of all previous nodes get updated according to (1) and (2) in Appendix A. The penalties are adjustable. Higher penalties lead to more diverse routes but longer computation times.

To obtain our algorithm DFPN* for restrosynthesis, we use the DFPN-E algorithm by Kishimoto et al. [16] as a baseline. This algorithm uses a heuristic function that evaluates the *cost* of using a certain edge from a molecule to its child (i.e., reaction). We then apply a diversity-controlling strategy to it. More specifically, the single node we choose to disprove as part of our diversity-controlling strategy is a deepest reaction in the found route, i.e., a reaction reached through a longest path from the target molecule. By doing so, we force the algorithm to find shorter routes without destroying too many possible routes. The reason we choose a reaction rather than a molecule to disprove is that disproving a molecule leads to a disproof of a reaction anyway. For the same reason, we set $p_{\text{mol}}=0$.

## Results & discussion

In the wider CASP literature the number of solved molecules is often used as proxy to compare the quality of different CASP tools [9, 30, 39]. This metric is highly dependent on the underlying size and make-up of the catalog of (assumed) buyable building blocks and thus not easily comparable between publications. Additionally, the number of solved molecules can vary a lot between different sets of molecules and thus we limit our comparison to the algorithms tested here. Looking at the smaller search times between 60 s and 300 s our DFPN* implementation solves more molecules than the MCTS implementation we used (see Table 1 and Fig. 11 in Appendix D.5). The MCTS implementation is taken from [34, 40], adding minor modifications to adapt it for our use. For search times of 600 s to 1200 s we do not see a difference between our DFPN* implementation and the MCTS regarding the number of solved molecules with both algorithms converging to a maximum of 94%.

**Fig. 2:**
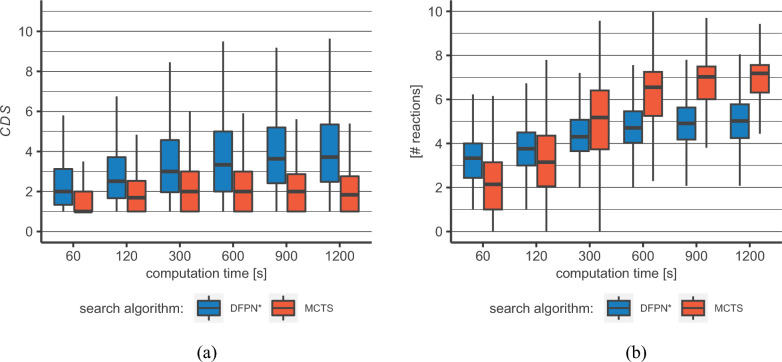
Comparison between DFPN* and MCTS: **a** Chemical diversity score *(CDS)* for DFPN* and MCTS routes for different search times. DFPN* routes show significantly higher diversity score throughout all data points (Asymptotic Wilcoxon-Mann-Whitney Test, significance level $\alpha=0.01$, colored boxes correspond to the IQR between the first and third quantile, whiskers go up-to/down-to 1.5 times of the upper/lower bound of the IQR). **b** For smaller search times (60 s/120 s) the mean number of used reaction over all routes and solved molecules by our DFPN* implementation is 3.5/3.9 vs. 2.6/3.5 for the MCTS implementation. For longer search times this trend reverses and the highest median number of used reactions seen for the DFPN* implementation is 5.1 vs 7.3 for the MCTS implementation (colored boxes correspond to the IQR between the first and third quantile, whiskers go up-to/down-to 1.5 times of the upper/lower bound of the IQR)

The second and main aspect we are focusing on is the chemical diversity of the generated routes. We do so by applying the chemical diversity score introduced above on the sets of routes from both approaches. Looking at Fig. 2a we see that for our MCTS implementation the median CDS, across all our tested search times, is never significantly higher than 2. According to our definition of the CDS this means that the median number of chemical ideas present in a given set of routes, irrespective of search time, is 2 for the MCTS. For our DFPN* implementation (see Fig. 2a) the median CDS for the smallest search time (60 s) starts at 2 and increases with search time significantly until 600 s where it tapers of into a plateau at 3.8 for 1200 s. We do not expect either of the two algorithms to reach significantly higher median CDS with even longer search times. Looking beyond the median CDS values we see that with our DFPN* implementation we can reach CDS around 9 compared to a CDS of 6 for the MCTS (top whisker corresponds to 1.5 times upper bound of IQR). The lower bound of the IQR of the DFPN* is always above the respective median MCTS CDS, and this gap becomes even more pronounced for search times of 900 s and 1200 s. The MCTS seems to iterate on the same chemical ideas with prolonged search times. This is further corroborated by the mean number of synthesis routes found (see Table 1). For the longer search times (starting with 300 s) the MCTS is able to find significantly more synthesis routes, but this is not translated into a set of more diverse routes (see Fig. 2a). This behavior coincides with our practical experience when working with routes found by our MCTS implementation and the results depicted in  Fig. 2b further strengthen this interpretation.

Finally, we want to move our attention to metrics describing the found pathways themselves. In Fig. 2b the mean number of reactions used in all pathways over all solved molecules is plotted against the search time for our MCTS and DFPN* implementation. For our smallest search time (60 s) DFPN* needs a median of 3.5 reactions to reach the target molecule. This rises slightly for search times of 600 s, where it then reaches a plateau for both of our highest search times 900 s and 1200 s. The standard deviation of the distribution for the DFPN* stays fairly constant over all search times and we see some outliers that need up to 11 reactions to reach the target. In comparison to that the MCTS needs, on average, less reactions for smaller search times (60 s and 120 s) i.e. 2.6 vs 3.5 reactions but then already surpasses the DFPN* with with a mean of 5.4 vs. 4.4 reactions at 300 s. We want to stress here that, while the mean number increases, there are still shorter routes present in the route sets stemming from longer search times. The trend observed in Fig. 2b comes from an increased length of those routes found additionally while the search progresses. The much slower increase of the mean number of reactions used by the DFPN* algorithm indicates that also in later stages of the search short routes are found, while the MCTS seemingly tends to further explore and deepen paths which already lead to presumably commercially available starting materials. This interpretation is also in line with the stagnation in the CDS of route sets produced by MCTS. The here reported results indicate that the MCTS implementation prioritizes shorter solutions early on, but is then modifying those only slightly at later stages of the search. The DFPN* algorithm does not have such a bias, yielding a mix of various route length from the beginning. But there are also algorithmically independent reasons for an increase in the mean number of reactions, true for both algorithms. One is of stochastic nature, as the number of possible solutions increases with each additional level of node depth in the search graph considered. Once most of the shorter and viable routes are found, increased search times for already solved molecules will naturally yield longer routes. Another reason for the observed trend lies in the increased number of solved molecules at longer search times. A reasonable assumption is that such molecules cannot be solved by short routes and require a more thorough exploration of the search graph, caused by the explosion of possible choices at deeper node levels. Since the number of additionally solved target molecules is low due to a high success rate early on and that such molecules also tend to have a smaller route set, their influence on the mean could be small. Therefore, the second effect might be less pronounced than the first.

**Fig. 3:**
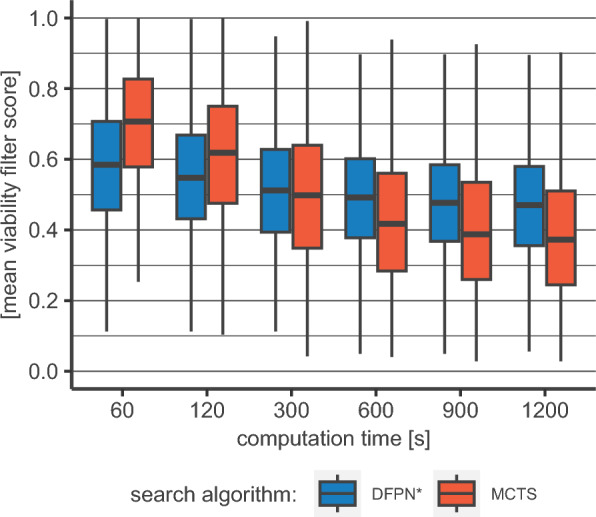
Mean multiplicative viability score for DFPN* and MCTS routes for different search times. The viability scores for each reaction of a route are multiplied with each other to give the estimated route viability. The respective mean score from each target molecule is given here. Higher values can be associated with a higher likelihood that the particular route will work in the lab. (Colored boxes correspond to the IQR between the first and third quantile, whiskers go up-to/down-to 1.5 times of the upper/lower bound of the IQR)

**Fig. 10:**
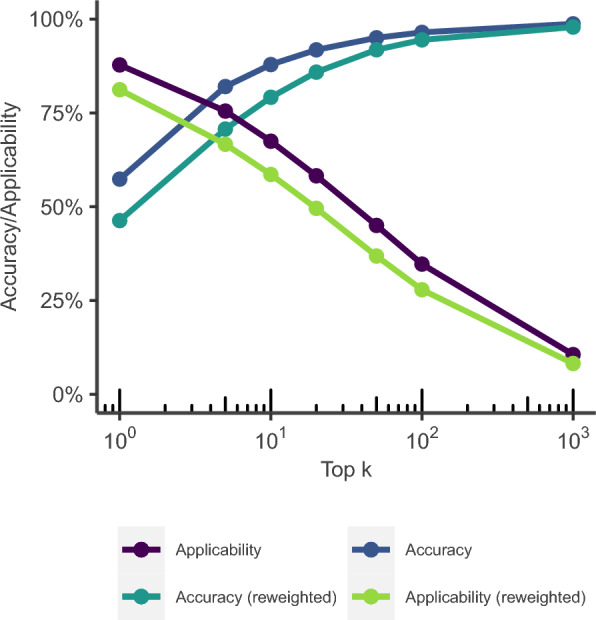
Top k accuracy and applicability for models trained on transformation classes reweighted according to their size, resulting in the same importance of all classes, and without such reweighting. Reactions found in in-house lab journals are stronger weighted in both cases and for the Applicability metric reactions were checked with our fast filter model

**Fig. 11:**
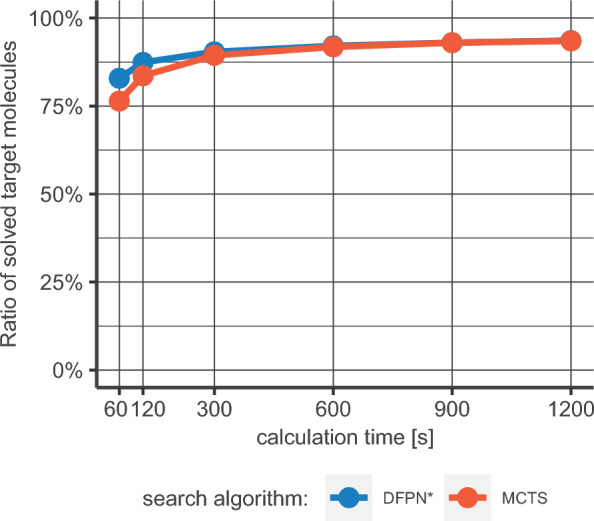
Percentage of solved molecules for the DFPN* and MCTS algorithms respectively. Shorter search times favor the DFPN* algorithm

**Fig. 12:**
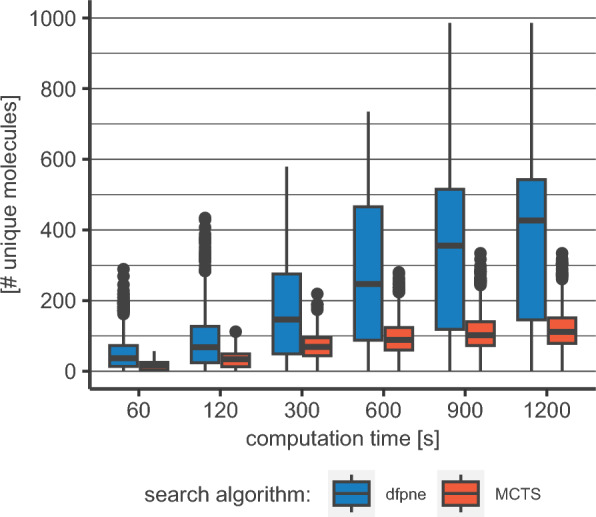
Number of unique molecules per Route. The differences of the medians for DFPN* and MCTS are statistically significant for all computing times (Asymptotic Wilcoxon-Mann–Whitney Test, significance level $\alpha =0.01$). We believe the much broader IQRs in the data generated by the DFPN* are a result of the higher diversity in said data. We assume a superlinear relation between CDS and the number of unique molecules, yielding wider IQRs than those found in the CDS analysis. Due to its mechanism of finding multiple routes via penalizing nodes on the search graph, the DFPN* algorithm is forced to explore a wider variety of molecules. (Colored boxes correspond to the IQR between the first and third quantile, whiskers go up-to/down-to 1.5 times of the upper/lower bound of the IQR, dots represent outliers.)

Routes can also be evaluated by the route viability. For this, we multiply the scores of our viability model for each reaction of a route with each other. The resulting score can be interpreted as the probability of the route working in the lab. This metric depends heavily on the predictions of said viability model. This is however not without caveats. The model is a binary classifier, discriminating between working and non-working reactions. But since there are only very few non-working reactions reported in literature, true negative data is very limited and is dwarfed by the bulk of true positive data. To overcome this imbalance, synthetically generated negative data is used [9]. Tested on proprietary in-house true and synthetic negative data, we found a large discrepancy between model performances, $\sim 65\%$ vs $\sim 95\%$ accuracy. Given also an unknown error to the in-house negative data (e.g., the reason why a reaction failed is often not known) we advice to be cautious when dealing with such scores in general. Despite its limitations, we nonetheless think that it can give valuable insights. In Fig. 3, the mean route viability is given for the DFPN* and MCTS algorithms. For shorter search times, the MCTS yields routes with an on average higher viability. At 300 s, the two implementations show very similar distributions and at search times beyond that, the DFPN* implementation yields higher viability scores. We attribute this, in parts, to the route-length distribution discussed above. Longer routes will often have lower viability scores due to the additional sub-one factors. This effect shows in the lower scores for our DFPN* implementation for shorter search times and also for the MCTS for longer ones. Another factor is the tendency of the MCTS to favor reactions with a high score assigned by the reaction prediction model. This steers it towards high-viability routes, but prevents it from creating meaningful diversity, as also shown above. The strategy for generating multiple solutions in our DFPN* implementation is designed to also pursue pathways with lower reaction prediction model scores. This is a conscious choice, using machine-learning models to search efficiently but not trusting them blindly. We believe the trade-off between estimated viability and diversity worthwhile.

**Table 1:** Fraction of molecules for which the individual algorithm was able to find at least one viable synthesis route and mean number of routes found for a given search time

Search time [s]	DFPN*	MCTS	DFPN*	MCTS
	Solved molecules [%]		Mean # of routes	
60	83	76	34.84	13.87
120	87	84	64.84	57.16
300	90	89	138.92	266.66
600	92	92	216.3	396.45
900	93	93	257.67	435.04
1200	94	94	284.21	445.04

## Conclusion

In this work, we adapted DFPN to find multiple solutions in the context of chemical retrosynthesis. In designing our changes to the algorithm, we made chemical diversity a priority, following the principle *quality through diversity*. We dubbed this new variant DFPN*. To quantify the diversity generated by DFPN*, we introduced a new chemical diversity score (CDS) that captures the number of unique chemical synthesis ideas present in a set of synthesis pathways. We compared DFPN* with MCTS on a diverse set of 1000 molecules extracted from the DUD-E database. Our DFPN* implementation shows comparable to slightly better performance regarding the number of solved molecules for all search time budgets. We also show that our DFPN* implementation is superior to the MCTS with regards to chemical diversity as well as to synthesis effort, measured by the mean number of reactions, at medium and long search time budgets.

Our approach tackles two major challenges in current CASP tools simultaneously. Currently available reaction data does not allow for near-error-free solutions and optimality in synthesis strategy is defined differently by the various branches of chemistry. The arguably best solution to both challenges is to provide a diverse set of pathways. From these, the users may choose those pathways that suit their needs best. We think that the DFPN* algorithm gives access to more user-oriented, higher quality CASP tools by proposing highly diverse synthesis pathways without compromising their individual quality.

The advancements in this work also give rise to further exploitation of the diversity principle. For example, from a set of pathways, calculated for a compound library, it is possible to search and select those that have the highest overlap in reactions, intermediates, and building blocks. Obviously, a high chemical diversity is critical to be able to find such a set. This approach would, on the one hand, give rise to a new compound selection criterion for experimental screening candidates and secondly could reduce the synthesis effort for the selected compounds significantly. Furthermore, computation is currently limited to a single core, and for single-target use cases no trivial parallelization scheme can be deployed, dictating wait times for users. Utilizing the multi-core design of modern processors would allow for a smoother integration in lab routines by shortening the time-to-solution.

## Figures and Tables

**Algorithm 1:**
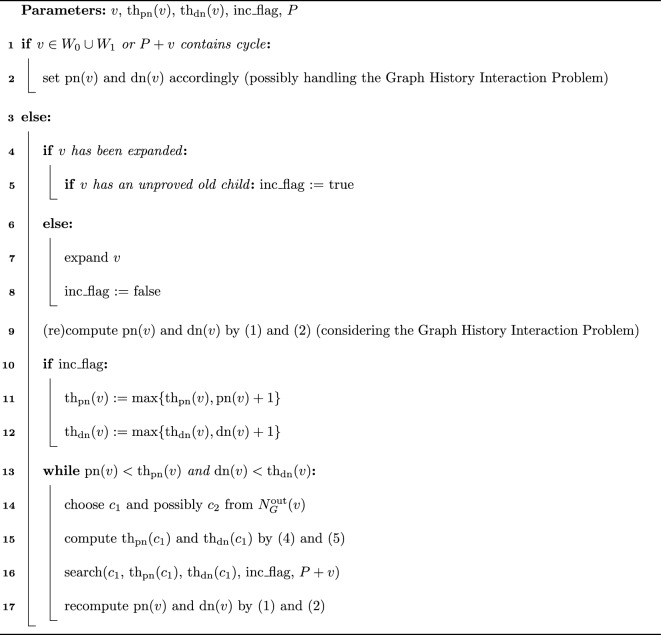
search (DFPN with TCA)

**Fig. 4:**
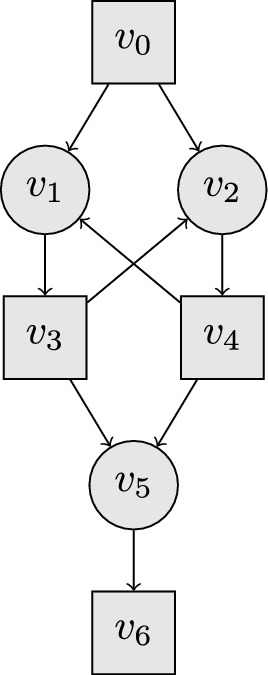
The graph *G* from Simpler counter example without TCA section in vertical orientation. Recall that the nodes in $V_0$ and $V_1$ are depicted as squares and circles, respectively, and that $W_0=\{v_6\}, W_1=\emptyset$

**Fig. 5:**
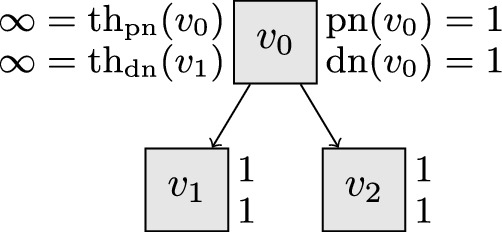
The explored part of *G* right after the expansion of $v_0$

**Fig. 6:**
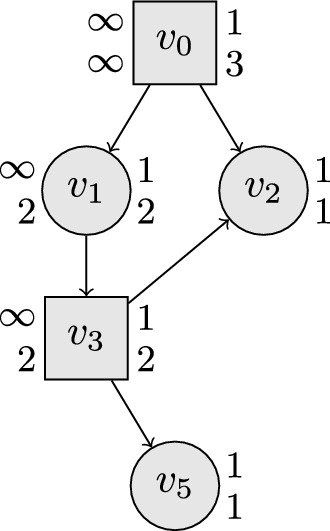
The explored part of *G* at the second visit to $v_0$

**Fig. 7:**
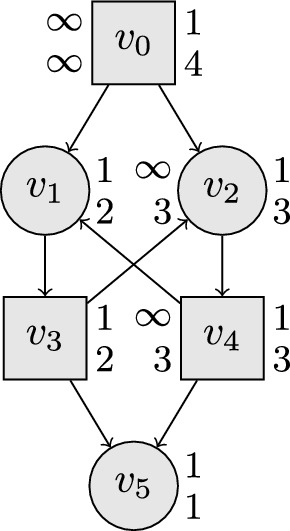
The explored part of *G* at the third visit to $v_0$

**Fig. 8:**
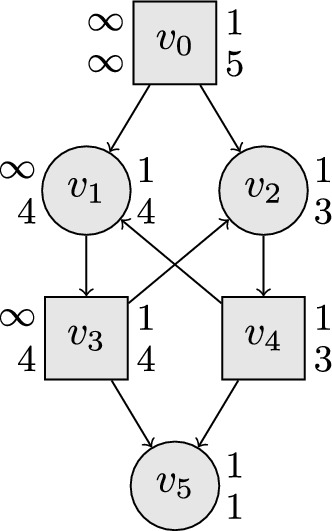
The explored part of *G* at the fourth visit to $v_0$

**Fig. 9:**
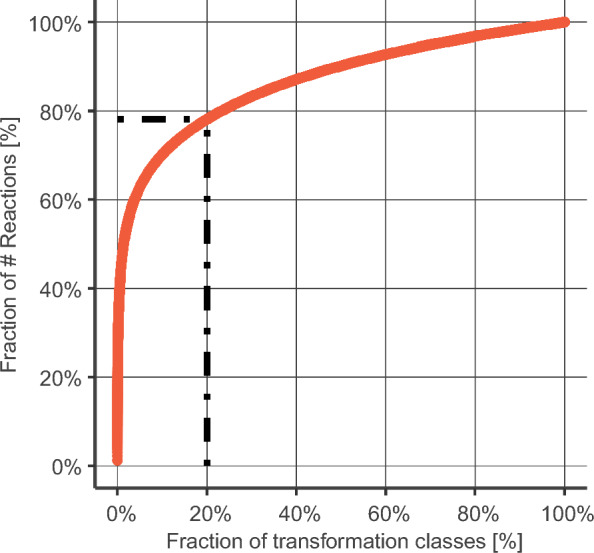
Fraction of the cumulative number of samples relative to the full data set (red)

**Fig. 13:**
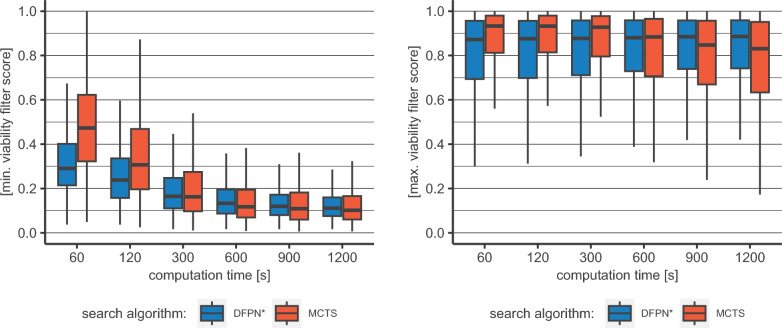
Comparison between DFPN* and MCTS with respect to the minimum and maximum route viability: (left) Minimum multiplicative viability score for DFPN* and MCTS routes for different search times. The viability scores for each reaction of a route are multiplied with each other to give the estimated route viability. The respective minimum score from each target molecule (i.e. the assumably worst route found) is distributed around lower values for the DFPN* than for the MCTS implementation for search times 60 and 120 s. For longer search times both algorithms converge to the approximately same level. (Colored boxes correspond to the IQR between the first and third quantile, whiskers go up-to/down-to 1.5 times of the upper/lower bound of the IQR). (right) Maximum multiplicative viability score for DFPN* and MCTS routes for different search times. The viability scores for each reaction of a route are multiplied with each other to give the estimated route viability. The respective maximum score from each target molecule (i.e. the assumably best route found) is distributed around lower values for the DFPN* than for the MCTS implementation for search times 60, 120, and 300 s. At 600 s the distribution is very similar, while for 900 and 1200~s the trend reverses. (Colored boxes correspond to the IQR between the first and third quantile, whiskers go up-to/down-to 1.5 times of the upper/lower bound of the IQR)

## Data Availability

The dataset that was used for the analysis presented in this paper is available as supplemental material

## Appendix

### A depth-first proof-number search and variants: formal description

**Basic Definitions.** The input graph is a directed graph $G=(V,E)$ where $v_0\in V$ is the start node. Further, *V* is partitioned into $V_0$ and $V_1$, the sets of nodes of Player 0 and Player 1 (corresponding to the molecule and reaction player, respectively), also referred to as AND and OR nodes, respectively. The sets $W_0$ and $W_1$, the sets of winning nodes of Player 0 and Player 1, respectively, are disjoint subsets of *V*. Without loss of generality, we assume that

- all neighbors of nodes in $V_0$ are in $V_1$ and vice versa.
- for all $v\in V$ it holds that $\delta _{G}^{{{\text{out}}}} (v) = \emptyset$ if and only if $v\in W_0\cup W_1$.

A *play* *P* is a sequence of $v_0,v_1,\dots ,v_k$ such that for all $i\in \{0,\dots ,k-1\}$, we have $v_{i+1}\in N^\text{out} _G(v_i)$. If *P* is a path in *G*, i.e., it contains each node at most once, we say *P* is winning for Player $i\in \{0,1\}$ if $v_k\in W_i$. If $v_0,\dots ,v_{k-1}$ is a path and $v_k\in \{v_0,\dots ,v_{k-1}\}$ (the last move closes a cycle), we assume the play is winning for either of the players, e.g., Player 0 wins or the Player *i* with $v_{k-1}\notin V_i$ wins [38].

A *strategy* for Player $i\in \{0,1\}$ is a function $\sigma :V_i\rightarrow V$ such that $\sigma (v)\, \in \,N_{G}^{{{\text{out}}}} (v)$ for all $v\in V_i$. A pair of strategies for both players naturally induces a play (that stops the first time it is winning for one of the players). For $i\in \{0,1\}$, we say that a strategy $\sigma _i$ for Player 1 is a *winning strategy* if $\sigma _i$ and each strategy $\sigma _{1-i}$ for Player $1-i$ induces a play that is winning for Player *i*.

**Proof-Number Search.** Proof-Number Search (PNS) [21] explores *G* starting from $v_0$, i.e., initially $v_0$ is *explored*, and all other nodes are not explored. No edges are explored at that time. PNS iteratively *expands* nodes *v* that were previously not expanded. This way, PNS learns if $v\in W_i$ for $i\in \{0,1\}$, and all edges in $\delta ^\text{out} _G(v)$ as well as *v*’s children $N^\text{out} _G(v)$ are explored. Note that, since *G* is not necessarily a tree, such endpoints have possibly been explored prior to the expansion of *v*.

A node *v* is called *proved* if a winning strategy for Player 0 can be inferred from the explored part of *G*; if a winning strategy for Player 1 can be inferred, it is called *disproved*. A node that is neither proved nor disproved is said to be *unproved*.

The *(dis)proof number* of an explored node *v* is defined to be the minimum number of explored unproved nodes that have to be (dis)proved for *v* to be (dis)proved. Clearly, the (dis)proof number of a (dis)proved node is 0; by convention the proof number of a disproved node and the disproof number of a proved node is defined to be $\infty$.

For any explored node *v*, PNS maintains estimates $\text{pn} (v)$ and $\text{dn} (v)$ for the proof and disproof numbers, respectively, of *v*. For any unexpanded node $v\in V$ (in particular, $v_0$ at initialization), PNS sets $\text{pn} (v)=1$ and $\text{dn} (v)=1$ in accordance with the definition of the actual proof and disproof numbers.

In each iteration, PNS selects a *most promising node* to expand. To do so, it first selects $v_0$. If the currently selected node *v* has already been expanded, PNS distinguishes two cases. If $v\in V_0$, PNS next selects a node *c* in $N^{{{\text{out}}}} (v)$ minimizing $\text{pn} (c)$. Otherwise, i.e., if $v\in V_1$, it selects a node *c* in $N^\text{out} _G(v)$ minimizing $\text{dn} (c)$. Once PNS has selected an unexpanded node, it expands this node.

After expanding a node, PNS first sets $\text{pn} (v)=1$ and $\text{dn} (v)=1$ for the newly explored nodes *v* (as described above). Then it updates $\text{pn} (v)$ and $\text{dn} (v)$ for all the nodes *v* selected between the expansion that just happened and the expansion before that, in reverse order of selecting. In particular, in case of the just expanded node, PNS may have learned that $v\in W_i$ for $i\in \{0,1\}$, or a cycle has just been closed. In accordance with the definition of the actual proof and disproof numbers, upon learning Player 0 wins, it sets $\text{pn} (v)=0$ and $\text{dn} (v)=\infty$; upon learning that Player 1 wins, it sets $\text{pn} (v)=\infty$ and $\text{dn} (v)=0$. In all other cases, for $v\in V_0$ it sets

1 $$\begin{aligned} \text{pn} (v):=\min _{c\in N^\text{out} _G(v)}\text{pn} (c);\;\;\text{dn} (v):=\sum _{c\in N^\text{out} _G(v)}\text{dn} (c), \end{aligned}$$

and for $v\in V_1$ it sets

2 $${\text{pn}}(v): = \sum\limits_{{c \in N_{G}^{{{\text{out}}}} (v)}} {{\text{pn}}} (c);\;\;{\text{dn}}(v): = \min _{{c \in N_{G}^{{{\text{out}}}} (v)}} {\text{dn}}(c).$$

If *G* is an out-tree, it is easy to see that $\text{pn} (v)$ and $\text{dn} (v)$ reflect the actual (dis)proof numbers. In a general graph, however, as can be seen, e.g., in the graph shown in Fig. 1, this is not necessarily the case.

**Depth-First Proof-Number Search.** Depth-First Proof-Number Search (DFPN) [18] does noFigat start the search for the most promising node from $v_0$ in each iteration. Instead, when node *v* is currently selected, it tries to use thresholds $\text{th}_{\text{pn}} (v)$ and $\text{th}_{\text{dn}} (v)$ to decide whether the path taken from $v_0$ to *v* is part of a path that PNS would take. In particular, if

3 $$\begin{aligned} \text{pn} (v)<\text{th}_{\text{pn}} (v);\;\;\text{dn} (v)<\text{th}_{\text{dn}} (v), \end{aligned}$$

then it continues the search like PNS would, and otherwise it backtracks one step. The values of $\text{pn}$ and $\text{dn}$ are only recomputed at the node that DFPN is currently considering.

The thresholds are determined as follows. Initially, $\text{th}_{\text{pn}} (v_0):=\infty$ and $\text{th}_{\text{dn}} (v_0):=\infty$. Suppose from node *v*, node $c_1$ is selected next, i.e., if $v\in V_0$ ($v\in V_1$), $c_1$ has the smallest value of $\text{pn}$ ($\text{dn}$) among $N^\text{out} (v)$. If $|N^{{{\text{out}}}} (v)| = 1$, the thresholds for $c_1$ are taken over from *v*. Otherwise, let $c_2$ be a node that has the smallest value of $\text{pn}$ ($\text{dn}$) among $N^{{{\text{out}}}} (v){ \setminus }\{ c_{1} \}$. If $v\in V_0$,

4 $$\begin{aligned} \text{th}_{\text{pn}} (c_1)&:=\min \{\text{th}_{\text{pn}} (v),\text{pn} (c_2)+1\},\nonumber \\ \text{th}_{\text{dn}} (c_1)&:=\text{th}_{\text{dn}} (v)-\text{dn} (v)+\text{dn} (c_1), \end{aligned}$$

and if $$v\in V_1$$,

5 $$\begin{aligned} \text{th}_{\text{pn}} (c_1)&:=\text{th}_{\text{pn}} (v)-\text{pn} (v)+\text{pn} (c_1),\nonumber \\ \text{th}_{\text{dn}} (c_1)&:=\min \{\text{th}_{\text{dn}} (v),\text{dn} (c_2)+1\}. \end{aligned}$$

We give the full algorithm, including the Threshold Controlling Algorithm, in Algorithm 1.

### B Missing material from Subsection Simpler counter example without TCA

In Fig. 4, we first repeat the graph from Simpler counter example without TCA section in the more common vertical orientation.

Recall that, in DFPN, initially only $v_0$ is explored. As a first step, this node is expanded. This leads to the explored part of the graph along with (dis)proof numbers and thresholds depicted in Fig. 5.

At that point, DFPN is indifferent between expanding $v_1$ or $v_2$ first. Suppose w.l.o.g. DFPN expands $v_1$ first. Note that, $\text{th}_{\text{pn}} (v_1)$ will remain $\infty$ while $\text{th}_{\text{dn}} (v_1)$ is set to 2. Since $v_1$ has only one child, its values of $\text{pn}$ and $\text{dn}$ will remain 1 even after its expansion, and the thresholds remain the same, implying that DFPN continues expanding at along this path to $v_3$. Since $v_3\in V_0$ has, however, two children, $\text{th}_{\text{dn}} (v_3)=\text{dn} (v_3)$ after the expansion of $v_3$. Hence, DFPN will backtrack back to $v_0$. The resulting explored part of *G* is shown in Fig. 6.

Now there is no indifference anymore and DFPN explores the nodes $v_2$, $v_4$ in a similar manner, eventually backtracking to $v_0$. The result is shown in Fig. 7. Subsequently, DFPN will again visits $v_1$, $v_3$ and backtrack to $v_0$; the result is shown in Fig. 8.

It is easy to see that this behavior will continue and DFPN will run into an infinite loop. Running DFPN with TCA on this example will probably help the reader’s understanding of DFPN with TCA and the fact that it might not have the same issue.

### C full proof of completeness of DFPN with TCA

The purpose of this section is to prove the following theorem.

#### Theorem 1

DFPN with TCA is complete.

In the proof, we assume towards a contradiction that DFPN with TCA runs into an infinite loop. First, we remove all the parts of *G* that are irrelevant during the infinite loop, obtaining a new graph $L=(V',E')$. More precisely, let $P^\star :=v_0,\dots ,v^\star$ be the largest path that is a prefix of *P* throughout the infinite loop. Then

- $V'$ contains all vertices in $P^\star$, all vertices visited during the loop, and all their children;
- $E'$ contains all edges in $P^\star$ and outgoing edges from vertices visited during the loop.

For instance, for the loop considered in Subsection Simpler counter example without TCA, *L* would consist of all nodes but $v_9$ and all edges but the one leading to $v_9$.

For a node, $v\in V'$, we now define its *level* $\ell (v)$ to be the length of the longest $v_0$-*v* path in *L*. Note that in the proof for DFPN on directed acyclic graphs [22], levels are also used but defined on *G* rather than *L*. Let $\ell _{\max }$ be the maximal level of any node in *L*. The fundamental property of *L* is the following.

Consider a call during the infinite loop at some node $n_c$ that eventually returns because the threshold condition is not met anymore, i.e., $\text{pn} (n_c)\ge \text{th}_{\text{pn}} (n_c)$ or $\text{dn} (n_c)\ge \text{th}_{\text{dn}} (n_c)$. Denote by *M* the set of nodes (including $n_c$) at which the threshold condition had been violated in the meantime.

#### Lemma 1

Consider some $m\in M$. There is no node $o\in V'\setminus \{m\}$ with $\ell (o)\ge \ell (m)$ such that there exists a path from *o* to *m* in *L*.

Note that the statement is trivially true in acyclic graphs, in which an $v_0$-*o* path can always be extended by an *o*-*m* path to a longer path, but not in cyclic graphs. We provide a proof.

#### Proof of Lemma 1

Suppose such a node *o* exists, and denote by $P'$ the *o*-*m* path in *L*. First note that *o* must be visited during the infinite loop. The reason is that otherwise

- either *o* is part of $P^\star$, in which case the only $v_0$-*o* path in *L* (a prefix of $P^\star$) could be extended by an *o*-*m* path in *L*, contradicting $\ell (o)\ge \ell (m)$;
- or *o* is a child of a node visited during the loop with $N_{L}^{{{\text{out}}}} (o) = \emptyset$, contradicting the fact that $P'$ exists.

Next, note that for any node *p* in $P'$ (in particular *m*) it must hold that $\text{md} (o)<\text{md} (p)$ during the loop. This is because otherwise there must exist a node $p'$ on $P'$ with successor $p''$ on $P'$ such that $\text{md} (p')\ge \text{md} (p'')$. Upon visiting $p'$, TCA would then increase the thresholds. This already contradicts the assumption that DFPN with TCA is in an infinite loop because it would eventually make progress by either expanding an unexpanded node or finding a new cycle.

Now consider the $v_0$-*o* path $P''$ of length $\ell (o)$ in *L*. Since $\ell (o)\ge \ell (m)$, $P''$ cannot be extended to a $v_0$-*m* path by $P'$. The reason for that must be that $P''$ intersects $P'$ at some node $p_0$. By the same argument as above, applied to the $p_0$-*o* subpath of $P''$, it holds that $\text{md} (p_0)<\text{md} (o)$.

Hence, $$\text{md} (o)<\text{md} (p_0)<\text{md} (o)$$; a contradiction. $$\square$$

The remaining part of the proof is quite similar to the proof of completeness of directed acyclic graphs [22]. Indeed, we call a node $v\in V'$ *consistent* if it fulfills (1) and (2) (as equations rather than assignments) and *inconsistent* otherwise. We also define the *inconsistency tuple* to be $(N_{\ell _{\max }},N_{\ell _{\max }-1},\dots ,N_0)$ where, for $i\in \{\ell _{\max },\dots ,0\}$, $N_i$ is the number of inconsistent nodes at level *i* of *L*. We call *L* *i*-consistent if $N_{\ell _{\max }}=N_{\ell _{\max }-1}=\dots =N_{i}=0$.

Using Lemma 1, the proofs of the following two lemmata are now quite similar to the completeness proof on acyclic graphs [22].

#### Lemma 2

Let $n\in V'$, suppose *L* is $\ell (n)$-consistent, and $\text{pn} (n)< \text{th}_{\text{pn}} (n)$ as well as $\text{dn} (n)<\text{th}_{\text{dn}} (n)$ holds. If the algorithm now searches *n*, it will expand a node or find a new cycle before the call returns.

#### Proof

We first show that the algorithm will select a child $n_c$ of *n* for which the condition $\text{pn} (n_c)< \text{th}_{\text{pn}} (n_c)$ as well as $\text{dn} (n_c)<\text{th}_{\text{dn}} (n_c)$ will hold. If *n* has a single child, this is clear. Assuming $n\in V_0$ and letting $c_d$ be the second best child (called $c_2$ in (4) and (5))), we can compute

$$\begin{aligned} \text{th}_{\text{pn}} (n_c):=&\min \{\text{th}_{\text{pn}} (n),\text{pn} (n_d)+1\}\\ >&\min (\text{pn} (n),pn(n_c))=pn(n_c) \end{aligned}$$

and

$$\begin{aligned} \text{th}_{\text{dn}} (n_c):=\text{th}_{\text{dn}} (n)-\text{dn} (n)+\text{dn} (n_c)>\text{dn} (n_c), \end{aligned}$$

using that the threshold criterion is met at *n* and that *n* is consistent. The argument in case $n\in V_1$ is analogous.

We now claim that $\ell (n_c)\ge \ell (n)+1$. The reason is the following: Otherwise, *n* must be part of a $v_0$-$n_c$ path (because then that path cannot be extended by *n*). In particular, then there must exist an *n*-$n_c$ path in *L*. But then, on the emerging cycle, there must be a node $p'$ with successor $p''$ such that $\text{md} (p')\ge \text{md} (p'')$, prompting TCA to increase the thresholds upon visiting $p'$ and eventually make progress.

The claim now follows because the algorithm successively selects nodes with higher levels, eventually expanding a node. $\square$

For the next lemma, recall that a tuple $(a_1,a_2,\dots ,a_k)$ is lexicographically smaller than a tuple $(b_1,b_2,\dots ,b_k)$, denoted $(a_1,a_2,\dots ,a_k)<_{\text {lex}}(b_1,b_2,\dots ,b_k)$, if for some *i*, it holds that $a_1=b_1,a_2=b_2,\dots ,a_i=b_i$ and $a_{i+1}<b_{i+1}$.

#### Lemma 3

Suppose not all nodes in *L* are consistent. Denote by *T* and *U* the inconsistency tuples right before $n_c$ is searched and right after the call has returned, respectively. Then $U<_{\text{lex}}T$.

#### Proof

Let $T=(t_{\ell _{\max }},t_{\ell _{\max }-1},\dots ,t_0)$ and $U=(u_{\ell _{\max }},u_{\ell _{\max }-1},\dots ,u_0)$. We define $m^\star$ to be a node from *M* with maximum level. First note that, since by Lemma 1, for all nodes *o* with $\ell (o)\ge \ell (m^\star )$ and $m\in M$, there is no *o*-*m* path in *L*. Hence, such nodes *o* cannot become inconsistent by a visit to a node in *M*. For all $i>\ell _{\max }$, this implies $u_i=t_i$. Further, when $\text{pn} (m^\star )$ and $\text{dn} (m^\star )$ are recomputed, by Lemma 2, the formerly inconsistent node $m^\star$ becomes consistent, meaning $t_{\ell (m^\star )}>u_{\ell (m^\star )}$. $\square$

These lemmata allow us to prove the Theorem.

#### Proof of Theorem 1

Recall that $P^\star =v_0,\dots ,v^\star$ is the largest prefix that is part of *P* throughout the infinite loop. Consider some time during the infinite loop. If *L* is not yet $\ell (v^\star )$-consistent at that time, Lemma 3 guarantees that it will be $\ell (v^\star )$-consistent after finite time. At that time, Lemma 2 implies that the next time the algorithm searches $v^\star$ it will expand a node or find a new cycle before it returns, thereby making progress. That is a contradiction to the fact that the algorithm is in an infinite loop. $\square$

### D Methods

#### D.1 Target compound selection

All of our experiments are based on a subset of the DUD-E data set, a target data set designed to help benchmark molecular docking programs by providing challenging decoys [41]. Out of 22805 initial molecules, we extracted the subset of active molecules and calculated all-to-all RDKit-fingerprint Tanimoto similarities with the RDkit package [42] and discarded those molecules with a similarity greater than 50% for a total of 2580 molecules. We built our final set of molecules from 1000 randomly selected molecules having between 19 and 33 heavy atoms.

#### D.2 Synthesis pathway calculations

We calculated all synthesis pathways using our own DFPN* implementation and a modified MCTS implementation based on the ASKCOS code. For all searches we used the same set of template prioritization and reaction viability models (described in Appendix D.4). The reaction viability inclusion threshold was set to $> 0.5$, the maximum search depth was limited to 7 reaction steps from the target, and search was terminated prematurely if 500 routes were found. We set $\sigma = 3$ and $p_{\text{reac}}=10$ in our DFPN* calculations and we set the exploration constant of MCTS to 2 and its maximum branching factor to 50. All calculations used the same set of building blocks (see Appendix D.3). For comparison and further analysis we searched for 60 s, 120 s, 300 s, 600 s, 900 s, and 1200 s respectively.

#### D.3 Template creation/extraction and building block molecules

The retrosynthetic reactions, selected by the respective algorithm during route search, are applied to the target molecule as well as to all intermediates as reaction templates in form of reaction SMARTS. These templates are extracted from known chemical reactions (as reactions SMILES), in our case taken from the Reaxys^®^^1^ database and in-house lab journal data. The extracted reactions are first standardized with the Pipeline Pilot Node [43] “Standardize Molecule”, removing all stereo chemistry information from the SMILES, single atom fragments, and standardizing all charges while keeping the original charges of acids. In a second step the reactions were filtered for maximum molecule size of 128 heavy atoms and single product reactions (a limitation of both search algorithms) or more than 3 reactants. The latter was checked with RDKit [42] which thereby also doubles as a filter for SMILES interpretability. As building-block molecules we used a version of Bayer’s internal catalogs. To align those compounds with our templates, the corresponding molecules were standardized in the same way, omitting the filtering step on heavy atoms.

The filtered and standardized reaction where than atom mapped with first the Namerxn software by Nextmove [44] and those reactions which could not be mapped that way where treated in Pipeline Pilot with the “Add Reaction Mapping” with a maximum MCSS search time of 600 s and maximum time per mapping of 1800 s. The extraction of SMARTS patterns from the atom mapped reactions was done with the open source Python package RDChiral [45].

In total, there were 11,960,851 transformations extracted from the combined data sets, which were then grouped into 2,819,644 classes of identical SMARTS. Consequently, the largest transformation classes are encoding the most abundant reaction classes. In an effort to filter out transformations containing errors from the atom-mapping step and the SMARTS extraction process as well as to exclude questionable, and therefore seldom publicized chemistry, a cutoff of at least five class members was applied, yielding a final data set containing about 270,605 classes and 8,616,236 million samples.

The distribution of class sizes as well as the cumulative proportion of the total data set is given as a Pareto diagram in Fig. 9. As indicated by dashed lines, only 20% of all transformation classes cover already about 80% of the full data set and, shown in the inset, the biggest class is composed of about 100,000 members while the $100^{\text {th}}$ already only contains mere 6,000 samples, dropping fast in the two-digit numbers range. This testifies to the extreme imbalanced nature of the here employed data set used for training the one-step-retro model.

#### D.4 Single-step retrosynthesis (template prioritization + reaction viability)

When choosing which single reaction is the most appropriate for a single retrosynthetic step, we follow previous approaches for the prioritization and application of templates [9, 46].

During the exploration phase, the DFPN* algorithm often needs to further expand a node before determining whether it can be proved. In chemistry terms, this means that the selected molecule is neither a building block nor well known enough to be considered a building block. When this occurs, a *reaction prediction model* generates a list of *k* possible retrosynthetic transformations that could be applied to the current molecule. This neural network model is trained as classifier from literature (extracted from the Reaxys^®^ (https://www.reaxys.com/) database), and in our case from internal laboratory journals extracted transformation classes with the reaction product as input [47, 48]. In this publication we are using two models trained on the same data and with the same architecture but one with modified class weights in training and one without. The purpose of the reweighting is to counteract the severe imbalance between class sized in the training set. To do so, the weights are calculated by dividing the maximum class size by the individual class sizes, giving smaller classes a higher weight in comparison to larger ones. In the following, the model trained without the class-size dependent reweighting scheme is marked with a dagger symbol as $\text {template prioritization model}^{\dagger }$ or $\text {TP-model}^{\dagger }$ while the one with is simply referred to as TP-model. However, in the training of both models at least one class-reweighting scheme was applied which gave reactions from the in-house lab journals more emphasis. This was also done class-wise by using a factor of 10 for such reactions and assigning the mean weighting-factor over all contributing reactions to the individual classes. In the case of the TP-model both weights were multiplied. The architecture of both variants was the same, a MLP neural network with two hidden layers of size 1024, using ELU activation functions. As input the binary Morgan fingerprint ($\text {length}=2^{16}$, $\text {radius}=2$) of the product molecule was used. Before feeding the input to the model, a variance filter was applied to unify the fingerprints [9], resulting in an input dimension of $2^{14}$. The according thresholds were established based on an analysis over the training-set and can be found as part of the supporting information. The output layer had a size equal to the number of transformation classes (270,605) with a softmax activation function. The training was performed over 100 epochs (the final model was chosen based on validation loss from those 100 epochs) using the Adam optimization algorithm on a sparse categorical cross-entropy loss with a learning rate of $1\cdot10^{-5}$ and a batch size of 512. A dropout rate of 0.1 was sufficient to minimize overfitting.

Every transformation candidate generated by one of the TP-models is further tested in a forward direction. If the reaction prediction model determines that the target molecule R can be split into sub-molecules $\text{R}_1$ and $\text{R}_2$ via transformation T, a *fast-filter model* determines whether a transformation involving molecules $\text{R}_1$ and $\text{R}_2$ is likely to succeed. This fast filter model is a fully-connected, single-layer neural network with ReLu activation and Sigmoid output. Its inputs are the concatenation of the binary ECFP fingerprints (radius=2, features=8192) of molecules ${\text{R}_1}$ and ${\text{R}_2}$. This model is trained as a binary classifier for the task of deciding whether a transformation involving its input molecules is likely to be successful. For training the in Tensorflow 1.9.0 implemented Adam optimizer was used with a learning rate of $1\cdot10^{-4}$. This model is trained with a set of 8 M working reactions taken from from the Reaxys^®^ database and our internal laboratory journals [47, 48]. We used 136k negative and 608k working reactions from our internal laboratory journals and 12.8M generated algorithmically following the general approach by Segler et al. [9].

#### D.5 Single-step retrosynthesis model quality

A key element in the route search with the here used implementations of the MCTS and DFPN* search algorithms is the proposition of suitable chemical retrosynthetic transformations. Consequently, the quality of the deep learning model providing those is central for the capabilities of the algorithm to solve molecules and to find a diverse set of synthesis pathways.

The measured accuracy for the model trained on re-weighted class importance is significantly worse than the one trained without, as shown in Fig. 10. The larger gap in accuracy for smaller *k* between both approaches is qualitatively expected and a consequence of the effective removal of the heavy class sizes imbalance in the data set (see Appendix D.3) for the TP-model. For it the statistical advantage of predicting the largest classes is eliminated which subsequently leads to a smaller success rate. However, not predicting the associated label for a given molecule does not necessarily mean a bad prediction in the context of a retrosynthesis tool as the one used in this paper. For most molecules there are usually more than one way to synthesize it. This is most obvious for the de-protection reactions, which are the largest classes, as they are only a subsequent step in most cases and are not meant to build the actual chemical structure. The fact that the gap in accuracy between the two models closes with larger *k*, can therefore be interpreted as a result of an alternative transformation prioritization. Although the model assigns the given label in the dataset a lower importance, it is still capable of finding it (in light of the total number of transformation classes of roughly 270,000, finding the assigned label within the first 1000 highest ranked classes is still a significant enrichment. Furthermore, even those can still be used in pathways in our implementations).

A similar argument can be made for the applicability of the transformations, where the best ranked transformations of the model trained on re-weighted classes show a slightly worse performance. With the statistical advantage of the bigger (and more general) classes removed, more specific transformations get predicted with a higher probability, leading to a drop in applicability (Figs. 11, 12, and 13) (Tables 2, 3, 4, and 5).

**Table 2:** Fraction of molecules in the test set for which the individual algorithms were able to find at least one synthesis route. The dagger symbol denotes experiments conducted with the transformation prediction model trained without the class size correcting weights on the loss function

Search time [s]	DFPN*	$\text {DFPN*}^{\dagger }$	MCTS	$\text {MCTS}^{\dagger }$
	Solved molecules [%]			
60	83	84	76	78
120	87	87	84	84
300	90	90	89	89
600	92	93	92	91
900	93	94	93	93
1200	94	95	94	94

**Table 3:** Median/Maximal Chemical Diversity Scores over all solved molecules: The DFPN* algorithm generates significantly higher CDSs than the MCTS could achieve. Additionally the data shows a slight advantage of the TP-model over the regular TP-model^†^ in both algorithms

Search time [s]	DFPN*			$\text {DFPN*}^{\dagger }$			MCTS			$\text {MCTS}^{\dagger }$		
	$\text {q}_{25}$	Median	$\text {q}_{75}$	$\text {q}_{25}$	Median	$\text {q}_{75}$	$\text {q}_{25}$	Median	$\text {q}_{75}$	$\text {q}_{25}$	Median	$\text {q}_{75}$
60	1.3	2.0	3.1	1.0	2.0	3.0	1.0	1.0	2.0	1.0	1.0	2.0
120	1.7	2.5	3.7	1.5	2.3	3.7	1.0	1.7	2.5	1.0	1.5	2.0
300	2.0	3.0	4.6	1.8	2.9	4.2	1.0	2.0	3.0	1.0	1.8	2.8
600	2.1	3.4	5.1	2.0	3.2	4.7	1.0	2.0	3.0	1.0	1.8	2.6
900	2.4	3.7	5.4	2.0	3.4	4.9	1.0	2.0	2.9	1.0	1.7	2.5
1200	2.5	3.8	5.6	2.2	3.5	5.0	1.0	1.8	2.8	1.0	1.7	2.5

**Table 4:** Median/Maximal number of unique molecules used per Route: DFPN* clearly uses more unique molecules during route search, which is an indication that the found routes are more diverse

Search time [s]	DFPN*			$\text {DFPN*}^{\dagger }$			MCTS			$\text {MCTS}^{\dagger }$		
	$\text {q}_{25}$	Median	$\text {q}_{75}$	$\text {q}_{25}$	Median	$\text {q}_{75}$	$\text {q}_{25}$	Median	$\text {q}_{75}$	$\text {q}_{25}$	Median	$\text {q}_{75}$
60	26.0	46.0	79.0	26.0	49.0	83.0	13.0	20.0	28.0	14.0	21.0	29.0
120	42.0	78.5	138.0	40.0	83.0	146.5	25.0	39.0	52.0	23.0	38.0	50.0
300	75.0	164.0	292.3	73.0	165.0	318.0	53.0	77.0	100.0	48.0	68.0	92.8
600	116.0	278.0	474.0	109.0	287.5	446.8	69.3	94.0	127.0	61.0	86.0	112.0
900	158.3	390.0	522.0	150.0	379.5	508.0	79.0	107.0	144.8	68.0	95.0	125.5
1200	188.8	448.0	550.5	184.0	425.5	531.8	85.0	116.0	154.0	77.0	103.0	135.0

**Table 5:** Median/Maximum of the mean number of reactions used for a set of routes per molecule: For shorter search times DFPN* uses more reactions to reach the target, than the MCTS. Around 300 s search time the picture reverses and the DFPN* uses less reactions to reach a target than the MCTS

Search time [s]	DFPN*			$\text {DFPN*}^{\dagger }$			MCTS			$\text {MCTS}^{\dagger }$		
	$\text {q}_{25}$	Median	$\text {q}_{75}$	$\text {q}_{25}$	Median	$\text {q}_{75}$	$\text {q}_{25}$	Median	$\text {q}_{75}$	$\text {q}_{25}$	Median	$\text {q}_{75}$
60	3.0	3.5	4.3	3.0	3.7	4.5	1.9	2.6	3.5	1.9	2.7	3.9
120	3.3	3.9	4.7	3.4	4.0	4.9	2.7	3.5	4.6	2.7	3.6	5.1
300	3.9	4.4	5.2	4.0	4.6	5.4	4.3	5.4	6.6	4.5	6.0	7.1
600	4.2	4.8	5.5	4.3	5.0	5.7	5.8	6.7	7.3	6.1	7.1	7.5
900	4.3	5.0	5.7	4.4	5.1	5.9	6.3	7.1	7.6	6.7	7.3	7.7
1200	4.4	5.1	5.9	4.5	5.2	6.0	6.7	7.3	7.6	7.0	7.4	7.7

The objectively worse performance of the TP-model in accuracy and applicability is not reflected in the number of solved molecules, as shown in Table 1, nor in the mean reactions per route ( Table 5) or the CDS ( Table 3). In fact, the opposite is the case, as the said model leads to slightly better results in both algorithms. These results attest to the limited expressiveness of the usually used metrics to evaluate reaction prediction models used in similar context. Although much more computationally expensive, testing the models as integrated parts of retrosynthetic route search algorithms seems to us as the only conclusive approach.

### D. 6 In-depth explanation of the *Chemical Diversity Score*

We now formalize the idea behind the CDS and give a descriptive explanation of our design choices. To do so, we take a target molecule R and a set *M* of *n* synthesis pathways $S_1,...,S_n$ to synthesize R, so $M=\{S_1,..., S_n\}$. To calculate the diversity of chemical ideas in *M* we propose the following procedure:

1. First, all bonds of R, which get formed in the different pathways, are identified. The set $\hat{S_i}$ contains all indices of bonds from R created during the synthesis with pathway $S_i$.
2. We denote a pathway $T \in M$ as *parent* of pathway $S \in M$ if $\hat{T} \subset \hat{S}$. Furthermore, pathways of *M* which do not have a *parent* in *M* are denoted as *core* pathways and the set of all core pathways of *M* we denote with $C_M \subset M$. In the case of $\hat{T} = \hat{S}$ only one of the both is in $C_M$.
3. Finally, with the *Jaccard distance* $d_J(\hat{T},\hat{T}'):= 1-\frac{|\hat{T} \cap \hat{T}'|}{|\hat{T} \cup \hat{T}'|}$ for two sets *T*, $T'$ and for $M\ne \emptyset$, the *Chemical Diversity Score* $\text {CDS}$ is defined as
  $$\begin{aligned} \text {CDS}:= 1 + \frac{1}{|C_M|} \sum _{T \in C_M}\sum _{T' \in C_M} d_J(\hat{T},\hat{T}') \end{aligned}$$

The $\text {CDS}$ can be interpreted as the number of different chemical ideas present in a given set of synthesis pathways. Higher $\text {CDS}$ values are considered better, as they indicate a higher diversity between them.

To perform step (1), all bonds present in the target molecules are indexed. In our case this is done with the RDKit on a SMILES-basis without explicit hydrogen. To identify the formed bonds in a pathway, the set of all bonds in R is compared to those present in the for this pathway used building blocks.

In step (2) pathways are selected, which cannot be represented by another, shorter one in the set. They represent the core of a chemical synthesis idea. The pathways that are not a member of $C_M$ can be seen as variations of them and contain additional, often unnecessary, steps for the synthesis of the target molecule R. In our understanding, those pathways do not contribute to the overall chemical diversity in a meaningful way.

In step (3) we compare the core pathways to each other, virtually building an all-to-all distance matrix using the Jaccard distance. The individual contribution of any pathway to the overall diversity and also to the $\text {CDS}$ can be obtained by the mean distances to all other pathways. The total $\text {CDS}$ is calculated by summing over those values for all pathways. Adding 1 sets the minimum value for the $\text {CDS}$, bringing it in line with the interpretation of the score as a measure of the number of chemical ideas in a set of synthesis pathways, which must be at least one, even if there is just a single pathway. The Jaccard distance is a value between 0 and 1. Pathways that create exactly the same bonds have Jaccard distance of 0, so they do not contribute to the overall diversity. For pathways that produce completely different bonds, we have $\hat{T} \cap \hat{T}'=\emptyset$ and therefore $J(\hat{T},\hat{T}')=1$.

If we now obtain different sets of synthesis pathways *M* and *N* for the same molecule R, e.g., from two different algorithms, we can now compare them in terms of diversity by calculating $\text{CDS}_M$ and $\text{CDS}_N$. This is possible since the CDS is intrinsically independent of the cardinality of the sets *M* and *N* themselves and focuses only on $C_M$ and $C_N$.
